# The Role of Risk Proximity in the Beliefs and Behaviors Related to Mosquito-Borne Diseases: The Case of Chikungunya in French Guiana

**DOI:** 10.4269/ajtmh.16-1028

**Published:** 2017-06-12

**Authors:** Claude Flamand, Camille Fritzell, Pauline Obale, Philippe Quenel, Jocelyn Raude

**Affiliations:** 1 Unité d’Épidémiologie, Institut Pasteur de la Guyane, Cayenne, France;; 2 Ecole des Hautes Etudes en Santé Publique, Université Sorbonne Paris Cité, Rennes, France;; 3 UMR Inserm 1085-IRSET Institut de Recherche sur la Santé, l’Environnement et le Travail, Rennes, France;; 4 UMR 190 EPV “Emergence des Pathologies Virales,” Aix-Marseille University, IRD 190, INSERM 1207, EHESP, Marseille, France;; 5 UMR “Processus Infectieux en Milieu Insulaire Tropical,” INSERM 1187, CNRS 9192, IRD 249, Université de La Réunion, Saint-Denis, France

## Abstract

Human behaviors are increasingly recognized to play a key role in the spread of infectious diseases. Although a set of social and cognitive determinants has been consistently found to affect the adoption of health protective behaviors aiming to control and prevent a variety of infections, little is currently known about the ecological drivers of these behaviors in epidemic settings. In this article, we took advantage of the outbreak of chikungunya, a reemerging mosquito-borne disease, that occurred in French Guiana in 2014–15 to test empirically the assumption proposed by Zielinski-Gutierrez and Hayden that the proximity of the disease and perceptions of the natural environment may considerably shape public response to an emerging health threat. To achieve this, a cross-sectional survey was conducted among high school students of the region (*N* = 1462) at an early stage of the epidemic. Surprisingly, spatial analysis of the collected data leads to counterintuitive results as the participants who lived in the most affected area expressed less concern about the disease and practiced preventive behaviors less frequently than did other participants. These paradoxical results may be attributed to the possible activation of risk denial processes which have previously been observed in the risk perception literature, and described by several social and psychological defensiveness theories.

## INTRODUCTION

Emerging and reemerging mosquito-borne diseases such as dengue fever, chikungunya, and zika represent a growing public health threat to tropical countries, especially American and Caribbean countries where a great number of large scale epidemics have been documented in recent years.^1,2^ In French Guiana, where the dengue virus has been responsible for several outbreaks over as many decades,^3–5^ a few locally acquired cases of chikungunya were reported in February 2014.^6^ Indeed, six biologically confirmed cases and 12 suspected cases were detected in Kourou municipality within a 200-m radius between February 19 and 27, confirming the first localized chain of chikungunya transmission in the American continent. The introduction and emergence of this new virus quickly triggered the implementation of various public health interventions including vector control, and awareness and prevention campaigns conducted by local health authorities. In the month following this first warning, the epidemiologic situation gradually evolved, as the number of clusters increased across the region.^7^ This led the public health authorities to implement the same control vector plan as for dengue fever.

In French Guiana, as in many tropical areas, prevention and vector-borne transmission reduction are largely based on an Integrated Vector Management strategy promoted by the World Health Organization and applied to all other vector-borne diseases.^8^ This strategy includes different approaches to tackle the local determinants of disease, including combining adequate environmental management aiming to reduce breeding sites, with the use of safe and effective insecticides, biological control using organisms that reduce the target species, education, and the promotion of personal protective behaviors.^9^

Community participation in vector control is now widely acknowledged as a key factor in the achievement and sustainability of these programs.^10,11^ One target group is young adults, as they may become deeply involved in community-based environmental management, and particularly in vector source reduction campaigns that aim to eliminate the most common breeding sites such as water containers.^11,12^ Similarly, the adoption of protective behaviors at an individual level, such as the use of insect repellent or mosquito nets, plays an important role in the prevention and control of infectious disease.^13^ However, although behavioral change is increasingly recognized as one of the biggest challenges to public health, we still do not know much about the cognitive, social, and ecological processes and factors that lead people to adopt effective protective behaviors in epidemic settings.

This is important as costly communication and educational efforts are often performed in tropical countries to reach vulnerable populations and to encourage them to adopt personal protective behaviors. In French Guiana, education campaigns have been implemented to improve awareness of the health threat posed by the proliferation of vectors in the home environment. Nevertheless, it remains unclear as to what extent the diffusion of this knowledge is associated with changes in beliefs, attitudes, and practices related to the prevention of vector-borne diseases.

Moreover, studies conducted on risk perceptions and behaviors indicated that both are likely to change significantly over time according to the occurrence of health events.^14–17^ Notably, the epidemiological trend may play a considerable role in the dynamic of beliefs, attitudes, and practices related to mosquito-borne diseases and their subsequent prevention. Therefore, it would be interesting to examine the perceptions and practices as a function of the proximity of the cases reported in the context of a novel vector-borne disease outbreak in a specific geographical area. Nevertheless, it should be noted that risk proximity is a multifaceted notion.^18^ It generally encompasses a temporal component (How imminent is the risk? How far away in time did the cases occur?), a spatial component (How close is the risk? How far away geographically did the cases occur?), and a sociocultural component (How personally relevant is the risk? How similar are the people affected in terms of cultural identity, values, or lifestyles?). In this article, we choose to use a somewhat narrow but common definition of the concept by focusing on the geographical or spatial proximity of the first cases of chikungunya to the individuals and their home communities.

### Theoretical background.

Today, there is still limited literature exploring the role of environmental or ecological factors, such as proximity to risk, on the adoption of health behaviors during outbreaks of infectious diseases. The literature explores to some extent the association between ecology and/or proximity and public perception of risk from various hazard sources, which include flooding, industrial facilities, and terrorism. In a pioneering study, Maderthaner and others^19^ examined the role of distance on risk perception. They found that increased spatial proximity to a range of public facilities tended to reduce the risk perceived by participants living in the vicinity of these potentially threatening objects. More recently, Rudisill and others^20^ found that living in increased proximity to avian flu was associated with a change in individual's consumption behavior. Fischhoff and others^21^ reported that living within 100 miles of the World Trade Center in New York City was associated with a higher risk perception of a subsequent terrorist attack. Similarly, Woods and others^22^ found a positive relationship between participants’ proximity to a potential terrorist target and their judgments of risk associated with a terrorist attack in their communities in the near future.

Therefore, the association between these ecological determinants (such as proximity or immediacy factors) and the adoption or change of health behavior may be mediated by another factor such as worry or risk perception. Indeed, the public’s perception of public health risks has long been of interest to the medical and public health community. It is considered to be a central construct of most health behavior theories or models,^23^ particularly cognitive models such as Health Belief Model and Protection Motivation Theory,^24^ as well as a reliable predictor of people’s likelihood to adopt protective behaviors.^25^

Nevertheless, research exploring the relationship between proximity and risk perception will be useful in supporting the clarification of the relationship between proximity and the adoption of health behaviors. In an article published in 2006, Zielinski-Gutierrez and Hayden proposed a stimulating model for defining risk perception of vector-borne infections, based on people’s appraisal of ecological conditions and diseases proximity, and developed from qualitative studies conducted in Colorado in the aftermath of the West Nile virus outbreaks that occurred in the region in the early 2000s.^26^ During the last decade, the introduction of geographic proximity to disease as an explanatory variable in quantitative studies designed to explain individual and cultural variations in protective actions taken in response to emerging health threats, led to inconclusive evidence in the empirical literature. Thus, spatial proximity to risk was found to have a limited influence on the consumption behavior reported by a large sample of European citizens to reduce the risk of contamination during the avian influenza outbreak that hit numerous countries in Spring 2006.^20^ In the same vein, Trumbo and Harper^27^ did not find that the perceived proximity of West Nile virus in Colorado exerted a significant influence on the protective behaviors reported by the participants, after adjustment for potentially confounding variables. However, as noted by Rudisill and others,^20^ it remains highly plausible that the effects of risk proximity are mediated to a large extent by some cognitive variables, such as knowledge or perceived susceptibility to health risks. Currently, many studies have shown that a multiple and complex interplay between proximal and distal determinants is likely to play a role in behavioral change processes related to health maintenance or preservation.^28^

In French Guiana, the arrival of Chikungunya virus (CHIKV) in the territory provides a unique opportunity to investigate the influence of spatial proximity of the risk on beliefs and behaviors related to a vector-borne disease. To this end, we conducted a study among high school students to estimate the extent to which ecological factors are associated with the adoption of health protective behaviors, as well as the beliefs and attitudes associated with the disease and its mode of prevention. The design of this study was guided by Zielinski-Gutierrez and Hayden’s model of risk perception based on the ecology and proximity model, in addition to variables drawn from more conventional social psychological models of health protective behaviors, such as the Health Belief Model^29^ or the self-regulation model of health threat.^30^ This study should help to understand better the interaction between social, cognitive, and environmental factors that have been found to be associated with health protective behaviors performed by individuals and groups in response to emerging health threats.

#### Settings.

Chikungunya fever is an arboviral disease caused by CHIKV, an alphavirus transmitted to humans primarily via the bite of an infected *Aedes* mosquito.^31^ This is an acute infection with an asymptomatic incubation period lasting on average between 2 and 3 days. Clinical onset is abrupt with symptoms generally resolving within 7–10 days. Supportive care with rest is prescribed during the acute joint symptoms.^32^ The illness is usually self-limiting and resolves with time. Nevertheless, complications, and indeed chronic complications such as polyarthralgia, can occur.^33,34^ There is no specific treatment of chikungunya and no vaccine is currently available.^32^

Historically, CHIKV was first isolated in 1952 when the first outbreak occurred in Tanzania.^31^ Since 2004, it began to spread at an unprecedented rate and has been responsible for epidemics in Africa, Asia, as well islands in the Indian Ocean and Europe.^35^ Before December 2013, its transmission had not been documented in the Americas, despite annually reported imported cases and the presence of the main vectors *A. albopictus* and *A. aegypti*.^36–38^ In December 2013, autochthonous cases were detected in the French territory of Saint Martin and led to a rapid spread and local transmission of CHIKV in the Caribbean and the Americas, including French Guiana.^39,40^ In this way, CHIKV spread progressively, putting the Americas at high epidemic risk, as vast areas of the American region were infested with the competent vectors.

**Figure 1.**
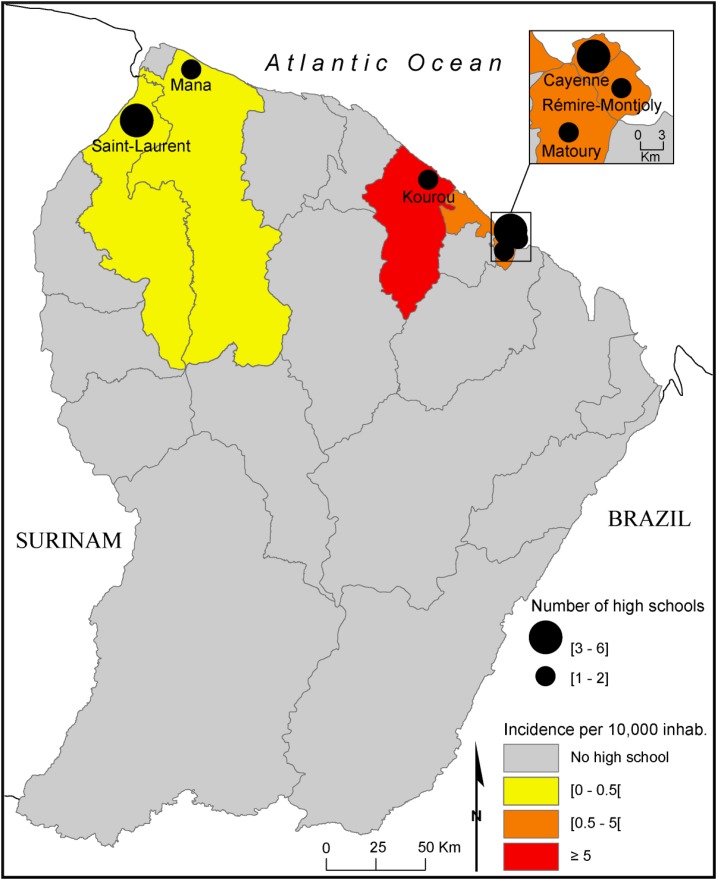
Geographic distribution of the chikungunya incidence and High School frequencies in French Guiana, May 2014. This figure appears in color at www.ajtmh.org.

French Guiana is an overseas department located on the northeastern coast of South America between Brazil and Suriname (Figure 1). At the time of the 2012 census, the population of the department was estimated at approximately 239,500 individuals. This region is characterized by an extremely high birth rate, and a high youth population, with 44% of the population being under 20 years old, and only 4% being older than 65 years old.^41^ In 2014, the territory boasted 15 high schools localized on the coastline in which 12,400 students were registered (approximately 5.2% of the whole population), and represented the target population of the study (Figure 1). At the beginning of May 2014, there existed a moderate level of autochthonous transmission in the region. Indeed, the regional epidemiology unit of the French national public health agency had detected 43 locally acquired confirmed cases from three different clusters.^42^ Almost half of these cases were located in the Kourou municipality, where the first and biggest cluster had been recorded in February. The control measures carried out under the epidemiological investigation of this cluster led to a mass media campaign about chikungunya prevention. A few days before the implementation of the survey, the cumulated incidence of chikungunya was at its highest in Kourou (7.70/10,000 inhabitants), whereas it was lower in the other areas (> 2.00/10,000 inhabitants) (Figure 1).

## METHODS

### Participants and procedures.

A cross-sectional survey about “beliefs, attitudes and practices” among students of French Guiana was conducted at the beginning of the epidemic, between the May 12 and 21 of May 2014, based on a randomized and stratified sample of French Guiana students, with the aim of investigating 1,600 students using a 2-stage selection procedure with 2.5% precision (95% confidence level). The population was first divided into two strata: high schools which provided both general and professional training, and professional high schools. In the second stage, among those two strata, 77 classrooms (second unit) were selected using simple random sampling in which each classroom represented a cluster. Finally, in each classroom sampled, all the students participated by responding to standardized, self-administered questionnaires under the supervision of local high school nurses. When the school did not have a nurse available, an investigator from the Pasteur Institute conducted the survey in these classes. Data were collected from the 200-items questionnaire.

The survey was carried out in accordance with the recommendations of the National Data Protection Authority (CNIL Declaration No. °aaH1243499A), which is responsible for ethical issues and protection of individual data collected in France. All of the information was collected anonymously, and an information letter was sent to parents through the students’ liaison notebook, informing them of the right to oppose the survey and to access the data. A signature was requested and checked by the school nurse before the survey was undertaken.

### Questionnaire and measures.

To measure the current proximity of risk within the context of this emerging health threat, the differences observed in the incidence rates among the main municipalities of the region were used as a proxy variable. The data concerning the spatial distribution of the cases of CHIKV infection were collected by the regional epidemiology unit of the French national public health agency through a passive surveillance system. The other measures were elicited through a questionnaire. Aside from the sociodemographic variables habitually recorded in this type of survey, the data were grouped into three general categories: 1) environmental variables that affect exposure to mosquito bites; 2) cognitive variables, in particular beliefs about the disease, and protective measures to prevent it; and 3) behavioral variables, notably the reported frequency of protective behaviors.

### Environmental and exposure variables.

The questionnaire contained a wide range of items such as the type of housing (“collective” if participants lived in a building containing multiple flats or “individual” if it were a house with only one household), the zone in which the participants lived and the presence or number of potential breeding sites or factors associated therewith, such as farming. In addition, respondents were asked how frequently they were bitten by mosquitoes (with response options: “Never,” “Seldom,” “Sometimes,” and “Often”). To complement this item, participants were also asked if they had ever seen, or heard about “*Aedes* mosquitoes,” how frequently they practiced outdoor activities and at what time during the day mosquito bites occurred. Participants were asked to report the occurrence of any acute febrile illness consistent with presumed dengue virus and/or CHIKV infections. The participants were also questioned about their exposure to messages provided by the public health authorities through various media (TV, radio, leaflets, social networks, and so on) to promote the adoption of health protective behaviors to prevent the risk of infection by the CHIKV (response options: “Yes,” “No,” and ”Unsure”; seven items, Cronbach’s α = 0.71).

### Cognitive and emotional variables.

A large range of beliefs were investigated, in particular perceptions of this health threat, that is, qualitative and quantitative judgments that individuals expressed when participants were asked to evaluate a specific illness, and the risk of contracting it.^43^ To characterize and evaluate these perceptions within the population, items were drawn from the existing literature by using the Brief Illness Perception Questionnaire. This questionnaire consists of eight items designed to identify rapidly and reliably a limited number of intraindividual determinants of particular behaviors related to health threats and illnesses in large-scale studies. These eight items are associated with the following dimensions^44^: “identity,” symptoms the patient spontaneously associates with the illness; “cause,” personal ideas about etiology; “timeline,” perceived duration of the illness; “consequences,” expected effects and outcomes of the illness; “treatment,” whether there are effective pharmaceutical products to enable recovery from the illness; perceived “control,” which refers to beliefs about whether the infection can be prevented or controlled by altering one’s behaviors; perceived coherence or “understanding,” the extent to which an individual believes he or she understands the illness and finally, the emotional response to the health threat, which was measured as a “feeling of worry” about the epidemic. Other items were drawn from the “Health Belief Model,” and adapted from the methodological literature devoted to transmissible infectious diseases, to assess perceived “exposure,” perceived “severity,” and perceived “susceptibility.”^45^ A number of items were also introduced in the questionnaire to assess participants’ understanding of the most common symptoms (five items, Cronbach’s α = 0.60) and how chikungunya is spread (three items, Cronbach’s α = 0.34). With the exception of the dimension associated with knowledge, cause, and identity of the illness, for which a binary response scale was used (1 = “yes”; 2 = “no”), respondents were asked to complete each item by grading it on an 11-point response scale (0–10) in which the meaning or value of each end point was indicated (for instance, from 0 = “not worried at all” to 10 = “extremely worried”).

### Behavioral variables.

In this section, participants were asked how often they undertook protective actions recommended by the public health authorities, such as wearing long-sleeved clothes, using repellent, or covering areas of water storages, by selecting one of the response options: “Never,” “Seldom, ” “Sometimes,” and “Often” (10 items, Cronbach’s α = 0.74). Then, participants were asked whether each of those behavioral recommendations were appropriate to prevent mosquito bites, by using a 5-point Likert scale (options: “Ineffective,” “Somewhat ineffective,” “Somewhat effective,” “Effective,” and “Not sure”; Cronbach’s α = 0.82). Finally, respondents were asked to assess the extent to which adoption of each protective measure recommended by the public health authorities to prevent chikungunya infection was constraining (response options: “Very constraining,” “Constraining,” “Somewhat constraining,” and “Not constraining at all”; Cronbach’s α = 0.76).

### Data analysis.

Data were recorded on Microsoft Access and statistical analysis was performed using STATA 12 software (Stata Corp., College Station, TX) and SPAD 12. Sampling weights were taken into account for all analyses to produce representative estimates. The first analysis aimed to explore the spatial variations in the beliefs, attitudes, and practices related to vector-borne diseases among students, by using descriptive statistical analysis. The sample was divided into three different groups to assess proximity to risk. The first group, called the “high proximity” group, included the Kourou municipality (*N* = 263) where the first epidemiological cluster of chikungunya diseases was observed, the second group, called the “intermediate proximity” group, included municipalities where the cumulated incidence was lower than 5/10,000 inhabitants (*N* = 804), and the “low proximity” group included the municipalities of Mana and Saint–Laurent, where only a few cases of CHIKV infection had been reported at the time of the survey (*N* = 296). The means or percentages (with 95% confidence intervals [CIs]) were calculated for each of the environmental, cognitive, and behavioral variables considered in the survey.

To identify sociodemographic, environmental, and cognitive factors associated with the level of self-reported protective behaviors, bivariate, and multivariate binomial logistic regressions were performed. The protective behavior, assessed by the frequency of adoption of personal actions reported by the participants in response to the risk of arboviral infection, was used as the dependent categorical variable (“Never,” “Seldom,” “Sometimes,” and “Often”). Factors proven to be significant in univariate analysis were tested in the stepwise multivariate model as independent variables. The level of statistical significance was set to 0.05.

## RESULTS

In total, 1,462 students took part in the study, including 225 participants (19.3% [95% CI 17.1, 21.8]) from the municipality of Kourou, where the first geographic clusters of cases of chikungunya were observed. No parental refusal was reported to the investigators. On the basis of the data collected among high school students of French Guiana, symptomatic chikungunya prevalence rates were estimated at 0.8% (95% CI [0.4, 1.6]) in May 2014. No cases of chikungunya were reported in the city of Kourou among the students living in this high-risk geographical area. However, 69 students (4.5%) were absent during the survey period, though there was no significant difference in absenteeism among the three groups. By contrast, it should be noted that a personal history of dengue fever infection was reported by 45.6% (95% CI [40.1, 51.2]) of the Kourou participants, which was comparable to that of students living in the other areas (44.3% [95% CI (40.5, 48.2)]), since geographical difference was not significant.

**Figure 2.**
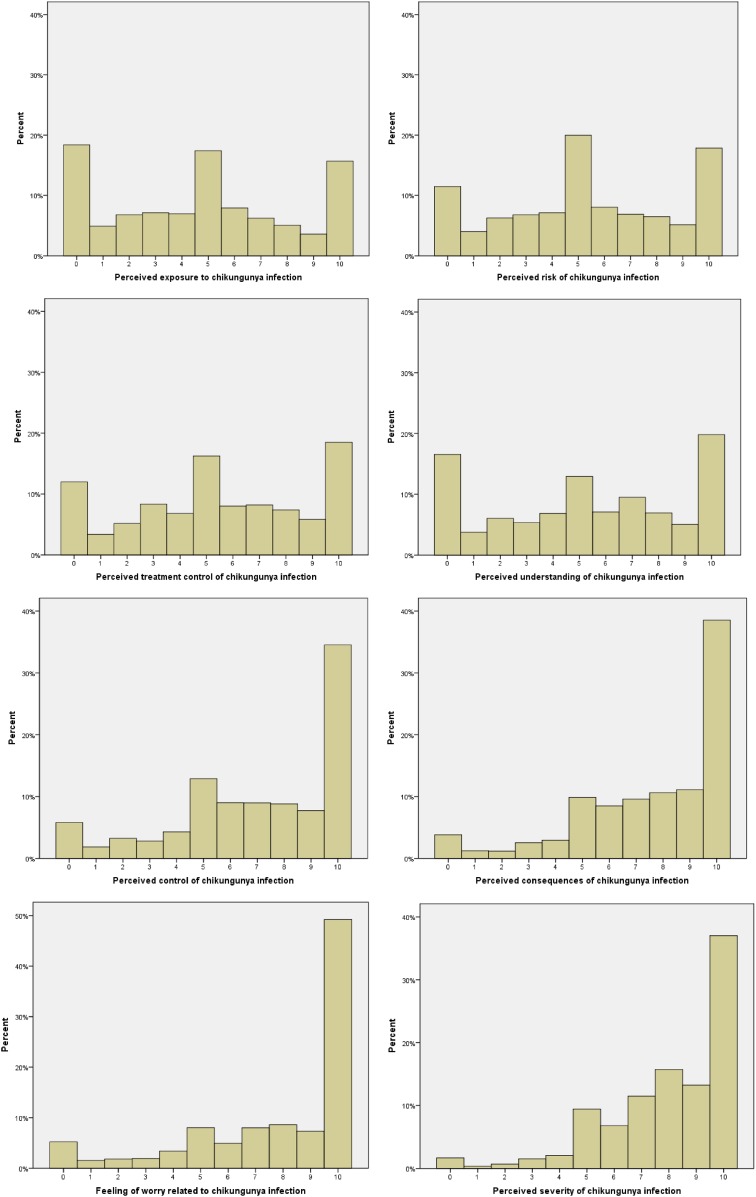
Distribution of the measures drawn from the “Brief Illness Perceptions Questionnaire” and the “Health Belief Model” on a 11-points Likert response scale (% of the observations). This figure appears in color at www.ajtmh.org.

**Figure 3.**
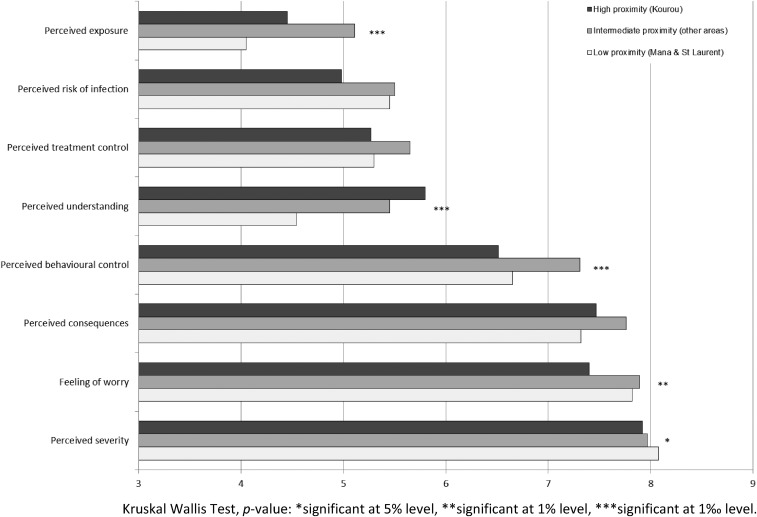
Perceived threat associated with chikungunya among students by their proximity to risk (arithmetic means). Kruskal Wallis Test, *P* value: * significant at 5% level, ** significant at 1% level, *** significant at 1‰ level.

The distributions of the main cognitive variables related to the chikungunya infection are displayed in Figure 2. Overall, chikungunya fever was perceived as being significantly more threatening than the other mosquito-borne diseases circulating in French Guiana. Thus, students were found to be more worried, to consider this disease more severe, and with more serious consequences than malaria, dengue, or yellow fever (Friedman’s *c*^2^ = 171 (3, 1,192), *P* < 0.001; 313 (3, 1,192) *P* < 0.001, respectively). Moreover, chikungunya disease was generally reported to be less well understood than dengue or yellow fever (Friedman’s *c*^2^ = 81 (2, 1,197), *P* < 0.001). Some significant spatial differences were however observed. As shown in Figure 3, students living in Kourou were significantly less worried about chikungunya than students living in other areas, with reported mean scores of 7.63 and 7.87, respectively (*t* (1,237) = −2.33, *P* = 0.025, *d* = 0.17). Similarly, the average perceived risk of infection was lower among Kourou high school students (*M* = 4.98 versus 5.49, *t* (1,183) = −2.18, *P* = 0.030, *d* = 0.16). In terms of perceived control, students living in Kourou considered chikungunya disease less controllable than other students, with mean scores of 6.51 and 7.12, respectively (*t* (1,187) = −2.91, *P* = 0.004, *d* = 0.21). Finally, regarding the level of understanding, students from Kourou reported a better understanding of chikungunya than did students from other areas, with reported mean scores of 5.80, and 5.20, respectively (*t* (1,200) = 2.43, *P* = 0.018, *d* = 0.17). This result is consistent with the observation that the Kourou participants were significantly more exposed to health messages about how to prevent CHIKV infection (*M* = 2.68 versus 2.39, *t* (1,362) = −2.13, *P* = 0.032, *d* = 0.15) than were participants in other areas.

The differences in the cognitive variables associated the mosquito-borne diseases and their mode of transmission between Kourou and other geographic areas are shown in Table 1. The table shows that high school students from Kourou had a better knowledge of the mosquito-borne disease vector than students living in other areas since 33.2%, as compared with 25.5% in the other communities, had already heard about “*Aedes*” (Pearson’s *c*^2^ = 4.78 (1, 1,415), *P* = 0.022), and 18.8%—as opposed to 13.2%—had actually identified these mosquitoes in their local environment (Pearson’s *c*^2^ = 6.66 (1, 1,317), *P =* 0.048). Moreover, the former were also more aware that only *Aedes* mosquitoes can transmit chikungunya than the latter, 70.5% among the Kourou participants and 62.8% among the other participants (Pearson’s *c*^2^ = 5.88 (1, 1,462), *P =* 0.046). Nevertheless, when asked about the typical symptoms associated to mosquito-borne diseases, the level of knowledge was found to be similar in both groups, with the notable exception of severe headache, which was less known in the Kourou community (69.7% as opposed to 78.1% elsewhere, Pearson’s *c*^2^ = 171 (3, 1,192), *P* = 0.004).

**Table 1:** Comparison of cognitive variables between students living in Kourou (high proximity), Mana and St. Laurent (low proximity), and other communities (intermediate proximity) answering “yes” (% of the observations [95% CI])

	High proximity	Intermediate proximity	Low proximity	*c*^2^ (df, *N*)	*P* value
*Aedes* observed	18.8 (17.7–20.2)	15.2 (10.9–20.6)	10.9 (8.8–13.4)	11.90 (2, 1,371)	0.003
*Aedes* known	33.2 (27.0–39.9)	26.5 (21.2–32.9)	23.0 (17.0–30.3)	7.72 (2, 1,415)	0.021
Mosquitoes transmit chikungunya	70.5 (64.8–75.6)	64.8 (59.0–70.1)	57.5 (51.8–62.9)	10.53 (2, 1,462)	0.005
Symptoms linked to chikungunya					
Headache	69.7 (59.1–78.5)	80.5 (76.7–83.8)	71.7 (62.7–79.4)	17.73 (2, 1,462)	0.000
Myalgia	72.0 (51.1–76.5)	73.3 (65.0–80.2)	63.5 (55.1–71.2)	10.14 (2, 1,462)	0.006
Arthralgia	54.0 (37.7–69.5)	61.7 (51.9–70.6)	40.3 (35.3–45.6)	28.23 (2, 1,462)	0.000
Asthenia	83.8 (70.5–91.8)	83.1 (78.9–86.5)	75.8 (66.5–83.2)	8.72 (2, 1,462)	0.013
Rash	35.0 (24.9–46.4)	36.5 (33.8–39.3)	36.7 (30.2–43.6)	0.24 (2, 1,462)	0.889

CI = confidence interval; *c*^2^ = chi square; df = degree of freedom.

The participants’ engagement in various health protective behaviors against mosquito-borne diseases is shown in Table 2. Although there were no differences observed among high school students from Kourou and those from the others areas in terms of perceived frequency of mosquito bites, students from Kourou were paradoxically found to engage in health protective actions less regularly than other students. Thus, 46.4% of the Kourou participants, compared with 54.1% of the other participants, reported adopting health protective behaviors aimed at reducing the risk of infection by mosquito-borne diseases (Pearson’s *c*^2^ = 5.11 (3, 1,192), *P* = 0.024). Overall, the most frequent preventive behaviors undertaken by the participants were the use of indoor insecticide sprays (67.2% [95% CI (64.8; 69.6)]), draining out stagnant water (60.4% [95% CI (57.9; 62.9)]), and closing doors and limiting outdoor activities (59.0% [95% CI (56.5; 61.5)]). When compared with the students from other areas, those from Kourou were less likely to use mosquito nets while sleeping and to cover water storage containers, with 20.5% and 41.8% for the latter, as opposed to 31.0% and 47.7% for the former, respectively (Pearson’s *c*^2^ = 11.24 [1, 1,364], *P* = .001; Pearson’s *c*^2^ = 5.24 [1, 1,364], *P* = 0.022).

**Table 2:** Comparison of protective behaviors between high school students living in Kourou, Mana/St. Laurent, and others areas (% of the observations [95% CI])

	High proximity	Intermediate proximity	Low proximity	*c*^2^ (df, *N*)	*P* value
Mosquito repellants	38.1 (34.0–42.4)	37.8 (34.4–41.3)	28.3 (25.1–31.6)	6.99 (2, 1,364)	0.030
Insecticide spray	60.7 (59.8–61.6)	66.6 (58.1–72.4)	68.9 (64.5–73.0)	3.35 (2, 1,364)	0.188
Vaporizer for insecticide indoor	24.2 (23.3–25.1)	22.1 (19.3–25.1)	30.2 (27.0–33.6)	8.16 (2, 1,364)	0.017
Vaporizer for insecticide outdoor	32.6 (26.4–44.3)	32.7 (27.2–38.7)	31.7 (22.9–42.1)	0.19 (2, 1,365)	0.906
Mosquito nets while sleeping	15.9 (11.7–20.0)	17.0 (14.4–20.0)	49.0 (39.6–58.4)	103.1 (2, 1,364)	0.000
Mosquito nets at windows	43.2 (36.4–50.3)	35.7 (32.4–39.2)	32.6 (28.5–37.0)	4.96 (2, 1,364)	0.084
Drain out stored water	57.3 (35.2–64.2)	56.5 (50.6–62.7)	57.6 (45.1–69.3)	0.03 (2, 1,364)	0.986
Cover storage containers	41.8 (39.5–43.0)	43.0 (36.9–49.3)	54.3 (45.0–63.3)	6. 73 (2, 1,364)	0.035
Close the doors, avoid outdoor activities	56.4 (43.4–68.6)	58.4 (53.1–63.5)	48.6 (44.8–52.3)	7.49 (2, 1,364)	0.024
Wear full-sleeved clothes	42.3 (37–47.8)	41.4 (35.2–47.9)	51.4 (38.8–63.8)	7.13 (2, 1,365)	0.028

CI = confidence interval; *c*^2^ = chi square; df = degree of freedom.

The unadjusted and adjusted odds ratios (ORs) resulting from the regression analyses and their significance are shown in Table 3. Findings from multivariate regression indicated only a few variables were associated with the adoption of protective behaviors against mosquito-borne diseases. After adjusting for potential confounding factors, two different types of determinants were found to be significantly associated with practices of protective behaviors among high school students. The first category refers mainly to ecological variables, since the participants’ place of residence and type of housing were found to relate to the adoption of health protective behaviors. However, it should be noted here that students who lived in Kourou were, even when adjusted for potentially confounding socioeconomic factors, less likely to report protective behaviors than students from other communities (adjuster OR [AOR] = 0.72; *P* = 0.006). The second category refers to the positive association between the self-reported adoption of protective behaviors and cognitive variables, namely knowledge of the *Aedes* mosquito and the perceived effectiveness of-protective actions recommended by the public health authorities.

**Table 3:** Association between sociodemographic, ecological, and cognitive characteristics, and protective behaviors reported by participants (ORs [95% CI], and *P* value)

Factors	Univariate model		Multivariate model	
	Unajusted OR	*P* value	Adjusted OR	*P* value
History of dengue fever	0.78 (0.77–1.39)	0.789		
History of chikungunya	0.80 (0.31–2.08)	0.632		
Risk proximity				
High proximity	Referent		Referent	
Intermediate proximity	1.21 (0.99–1.49)	0.061	1.19 (0.97–1.45)	0.080
Low proximity	1.66 (1.20–2.28)	0.004	1.61 (1.13–2.31)	0.012
Gender				
Female	Referent			
Male	1.03 (0.83–1.29)	0.724		
Type of high school programs				
Academic	Referent			
Apprenticeships	1.09 (0.81–1.47)	0.527		
Level of parent education				
Primary school	Referent			
Some secondary school	1.28 (0.83–1.97)	0.241		
Completed high school	1.53 (1.00–2.34)	0.046		
Some college and higher	1.30 (0.85–1.99)	0.202		
Household size				
1–2	Referent			
3–4	1.11 (0.84–1.46)	0.426		
5–6	1.03 (0.86–1.25)	0.676		
7 and more	1.47 (1.08–2.00)	0.016		
Type of housing				
Collective	Referent		Referent	
Individual	1.46 (1.18–1.82)	0.001	1.35 (1.07–1.71)	0.014
Zone				
Rural	Referent			
Half urban	0.70 (0.47–1.06)	0.092		
Urban	0.93 (0.69–1.25)	0.629		
Presence of yard	1.18 (0.95–1.46)	0.116		
Precence of pool	1.16 (0.91–1.48)	0.209		
Air conditionner	0.92 (0.70–1.20)	0.525		
Farming	1.07 (0.72–1.58)	0.708		
Presence of animals	0.83 (0.66–1.06)	0.130		
Frequency of mosquito bites	0.98 (0.74–1.30)	0.929		
Observation of *Aedes* mosquito	0.86 (0.67–0.94)	0.260		
Knowledge of *Aedes* mosquito	0.80 (0.08–0.56)	0.012	0.75 (0.61–0.92)	0.008
Perceived level of information	1.20 (0.96–1.51)	0.100		
Perceived cause	0.97 (0.79–1.20)	0.820		
Perceived worry				
< 4	Referent			
4–7	0.78 (0.59–0.98)	0.048		
> 7	0.97 (0.65–1.44)	0.880		
Perceived control				
< 4	Referent			
4–7	1.07 (0.74–1.55)	0.691		
> 7	1.16 (0.82–1.62)	0.368		
Perceived severity				
< 4	Referent			
4–7	0.71 (0.42–1.19)	0.187		
> 7	0.92 (0.59–1.44)	0.714		
Perceived consequences				
< 4	Referent			
4–7	0.74 (0.53–1.03)	0.073		
> 7	0.94 (0.54–1.00)	0.050		
Perceived exposure				
< 4	Referent			
4–7	0.86 (0.67–1.09)	0.210		
> 7	0.88 (0.68–1.14)	0.339		
Perceived treatment control				
<4	Referent			
4–7	1.02 (0.85–1.23)	0.786		
> 7	1.15 (0.91–1.45)	0.200		
Perceived understanding				
< 4	Referent			
4–7	1.05 (0.70–1.59)	0.771		
> 7	1.28 (0.85–1.91)	0.209		
Perceived risk of infection				
< 4	Referent			
4–7	0.91 (0.74–1.12)	0.367		
> 7	0.95 (0.74–1.23)	0.719		
Perceived efficacy of several means				
Not effective	Referent			
Somewhat effective	1.81 (1.34–2.43)	0.001	1.86 (1.39–2.49)	< 0.001
Effective	2.48 (1.97–3.14)	< 0.001	2.64 (1.99–3.50)	< 0.001

CI = confidence interval; OR = odds ratio. Unadjusted and adjusted odds-ratios, and *P* value were calculated from ordinal regression models.

## DISCUSSION

Though behavioral changes in response to threatening events in modern societies were largely ignored for decades in the epidemiological literature, they have recently become the focus of a range of research studies. Interestingly, health and social behaviors are now increasingly recognized as playing a fundamental role in the emergence, spreading, and persistence of infectious diseases in the world.^46,47^ This is particularly true in the field of vector-borne diseases, for which active participation of communities and alteration of human behaviors at a local level have long been thought to be critical in the implementation of effective and sustainable programs of vector control aiming to reduce the risk of infection with mosquito-borne viruses.^9–11^ However, it should be acknowledged that much is still unknown about the determinants and drivers of health protective behaviors in epidemic settings. Indeed, the most important findings about health behaviors and their determinants are currently drawn from empirical studies conducted in the domain of chronic or degenerative diseases, such as cancers, diabetes, or cardiovascular diseases. To date, there has been little attempt to measure the cognitive and behavioral responses to a vector-borne disease immediately after its emergence in a naïve population. Therefore, we cannot exclude that there may exist substantial differences between the nature and drivers of health behaviors engaged in nonstressed environments, as compared with those activated in stressed environments.^48^

In the context of French Guiana, we took advantage of an outbreak of chikungunya to attempt to investigate with greater accuracy the influence of distance on lay cognitive and behavioral responses to an emerging health threat. As noted in the introduction, we tested the hypothesis that an individual’s knowledge, beliefs, and practices related to chikungunya and its prevention vary as a function of the geographical proximity to the disease. For instance, it could be legitimately expected from a rational actor perspective that the likelihood of taking personal protective actions increases when the risk of contracting chikungunya is higher, due to the spread of the disease in a specific community or geographic area.

Overall, with a few exceptions, the empirical results collected among high school students during the emergence of the epidemic did not confirm our assumptions. In particular, families residing in the Municipality of Kourou were, surprisingly, found to report fewer protective actions than did families from the other areas, although the incidence rate of chikungunya was substantially higher in Kourou. As shown in Table 2, most of the protective behaviors recommended by the public health authorities were less frequently adopted in this high-risk area. Nevertheless, it should be noted that such findings appear somewhat consistent with prior evidence that participants living close to hazardous facilities are prone to report less concern about potential hazards than those living at a greater distance from these facilities.^49^ In other words, it seems easier for individuals to alter their beliefs and feelings about a possible health threat than to change their place of residency. This trend can also be observed in the case of the current chikungunya outbreak, as respondents living in Kourou were found to be slightly less worried about the risk of infection than were the other participants (*M* = 7.63 versus 7.87 (*t* (1,237) = −2.33, *P* = 0.025, *d* = 0.17). Interestingly, the geographic proximity to risk was also found to impact perceived behavioral control over the chikungunya infection. This result is important since perceived control has been documented as playing a crucial role in behavioral change in response to various health threats, particularly in health behavior research based on the Protection Motivation Theory or the Theory of Planned Behaviors.^23^

By contrast, it was expected that risk proximity would affect the individual’s level of knowledge about the disease and its vector of transmission. In this matter, our findings were rather congruent with those of recent research, as participants living in Kourou better understood how chikungunya was transmitted. Consistently, they also reported a better (subjective) understanding of the disease. As shown in Figure 3, the Kourou subgroup of students displayed a higher mean rating on the question asking them how well they thought they understood this illness. This spatial difference in knowledge and perceived understanding of chikungunya can be easily explained by the fact that greater proximity to a health threat is likely to motivate people to seek and process information about the disease and its prevention.^50^ However, as mentioned earlier, the community who engaged in more effortful information seeking and processing was surprisingly not found to be more predisposed to adopt health protective behaviors aiming to reduce the risk of infection.

This paradoxical finding raises a number of interesting questions. First of all, what are the underlying mechanisms that might help to explain these inconsistent results? Over the last decades, a variety of psychological and sociological investigations have been devoted to the so-called phenomenon of “risk denial.” This notion is derived from various theories of defensiveness, which hold that there is a tendency for high-risk groups of people to actively minimize or ignore the negative consequences of their risky behaviors or conditions on their health state and/or social participation.^50,51^ For instance, it has been repeatedly found that individuals who had experienced a particular health disorder tended to consider it less serious and more frequently occurring than individuals who had no such experience.^50,52^ In the case of an outbreak of vector-borne diseases, the proximity of the risk might activate a similar denial process, as the health threat may be perceived as less frightening and controllable by the population living in the most exposed area. Moreover, the data collected among high school students permitted the exploration of the motivational or cognitive nature of processes induced by risk proximity. Are these results really a manifestation of a defensive motivational response to threat, or are they caused by a more rational adaptation to risk? Indeed, research and theory from the risk perception literature have shown that the adoption of health protective behaviors leads to a reduced perceived risk associated with an epidemiological event.^22,53^ This risk reappraisal effect is the reason for which any correlation between risk-related judgments and behaviors is particularly difficult to interpret in cross-sectional studies.^†^ However, our survey demonstrated that the participants from Kourou were less likely to report preventive behaviors in response to the emerging health threat induced by the spread of chikungunya in their area. Consequently, even though the activation of other cognitive processes cannot be excluded, we believe that a form of defensive motivational process, like risk denial, appears to represent a better psychological explanation of these observations than a more rational process.

## LIMITATIONS

A number of possible limitations to our results should be reported. First, self-reports of preventive behaviors in surveys are known to have an uncertain relationship to the behaviors participants actually perform in their everyday life. Notably, self-reported behaviors in the health and safety domain are often subject to some biases attributable to the courtesy or social desirability effects. Nevertheless, there is no reason for assuming that this bias should be bigger in a low-risk area than in a high-risk area. Second, it is possible that some uninvestigated third factors, for example, a lower density of mosquito in Kourou, was responsible for the geographic differences observed in both beliefs and behaviors related to mosquito-borne diseases.^54^ Third, the survey was conducted among high school students who may have an understanding, attitudes, and perceptions that differ from those of the household members responsible for implementing control measures associated with vector-borne diseases. However, this target group, which was readily accessible and available, may represent a significant proxy for the household decision-makers. Furthermore, it was of interest to explore a young generation, which may be more easily involved in community-based vector source reduction campaigns. Last but not least, we cannot rule out the possibility that students absent during the survey period had been infected by CHIKV chikungunya. This may have led to an underestimation of the reported CHIKV chikungunya prevalence among students. Nevertheless, the overall epidemiologic situation, as reported by the public health authorities at the time of the survey, was characterized by a moderate autochthonous transmission and only 4.5% of the students registered in the surveyed classrooms were absent. Furthermore, we did not observe any significant differences in absenteeism between the various municipalities, which indicates that the interpretation of the results in terms of risk proximity is therefore unlikely to be a limitation.

## CONCLUSION

To conclude, exposure and proximity to risk induced by an outbreak of vector-borne diseases was found to lead to additional knowledge in the exposed population, but not necessarily to the acknowledgment that the risk of infection can be avoided or reduced. When groups of people are confronted with an emerging health threat, they may neglect the warning from the public health authorities to maintain a positive image of their community and health environment, notably by minimizing perceived control of and exposure to the risk of contracting the illness. This motivational process leads them to leave their former habits and lifestyles unchanged and, as a consequence, to increase their risk of infection from vector-borne diseases. Therefore, the psychological processes activated during outbreaks may impede to a large extent programs developed by public health institutions to control and prevent the propagation of vector-borne diseases in epidemic settings. Interestingly, this propensity to deny or neglect health risks is relatively well-documented at an individual level. In terms of practical implications, it seems advisable to offer at the community level a monitoring of health protective actions carried out at regular intervals to make the gap between the actual and the recommended behaviors more visible. In the past years, this strategy has been relatively successful to promote physical activity and healthier diet.^55,56^ Moreover, as social comparisons are known to support behavior change,^57^ feedback from such investigations could be communicated to the various communities of the regions concerned by an outbreak to show them some of the inconsistencies. In this way, it is expected that such a feedback loop would provide groups of people with an ongoing understanding of their own protective behavior and whether it matches and is appropriate to the environmental need, thereby allowing them to adapt preventive behaviors in a dynamic way, within a constantly evolving epidemiological environment.

## Footnotes

† As noted by Brewer and his colleagues (2004), the incorrect conclusion that higher perceived risk leads to less protective action can be drawn from statistical tests based only on cross-sectional data.
